# A Systematic Review of Aptamer‐Based Alginate‐Agarose Hydrogel Strategies for Inhibiting Keloid Fibrosis and Hypertrophic Scarring

**DOI:** 10.1111/jocd.71126

**Published:** 2026-10-12

**Authors:** Xiaorui Hu, Nur Azida Mohd Nasir, Shazana Hilda Shamsuddin, Nur Fatiha Ghazalli, Nurul Syazana Mohamad Shah

**Affiliations:** ^1^ Burn and Reconstructive Department General Hospital of Ningxia Medical University Yinchuan China; ^2^ Department of Plastic Surgery and Reconstructive Sciences School of Medical Sciences, Universiti Sains Malaysia Kota Bharu Kelantan Malaysia; ^3^ Pathology Department School of Medical Sciences, Universiti Sains Malaysia Kota Bharu Kelantan Malaysia; ^4^ Material and Chemical Unit School of Dental Sciences, Universiti Sains Malaysia Kota Bharu Kelantan Malaysia

**Keywords:** alginate‐agarose hydrogel, DNA aptamer, fibrosis inhibition, hypertrophic scar, keloid, molecular targeting

## Abstract

**Background:**

Abnormal wound healing leading to keloids and hypertrophic scars is driven by persistent fibroblast activation and excessive extracellular matrix accumulation, yet effective therapeutic options remain limited. Novel approaches, including nucleic acid aptamers and biomaterial‐based delivery platforms, have recently attracted attention.

**Objective:**

This study systematically reviews current research on the application of DNA aptamers and alginate–agarose hydrogels in fibrosis‐related conditions.

**Methods:**

Literature searches were performed in PubMed, Web of Science, and Scopus for studies published between January 2000 and March 2025. Studies meeting predefined criteria were grouped according to their primary focus on aptamer‐based targeting, hydrogel‐based delivery, or combined strategies. Due to heterogeneity, the included studies were synthesized descriptively.

**Results:**

Sixteen studies were included. Investigations of aptamers primarily addressed modulation of profibrotic signaling pathways, particularly those associated with TGF‐β and IL‐6. Hydrogel systems, especially alginate and agarose formulations, were mainly explored for their ability to prolong local drug retention and enable controlled release. Integrated approaches combining both strategies were rarely reported.

**Conclusion:**

Both aptamer‐based targeting and hydrogel‐mediated delivery demonstrate potential in fibrosis management. However, the predominance of preclinical evidence and the limited development of integrated systems indicate that further research is required to advance clinical translation.

## 1 Introduction

Pathological scarring, primarily manifesting as keloids and hypertrophic scars, represents a significant clinical challenge due to their complex fibrotic processes and high recurrence rates. These two major types of pathological scars share common features of excessive dermal fibrosis but differ in clinical presentation, histopathology, and biological behavior. Keloids typically extend beyond the original wound margins, do not regress spontaneously, and are characterized by aggressive fibroblast proliferation and abundant extracellular matrix (ECM) deposition. In contrast, hypertrophic scars are confined to wound boundaries, may regress over time, and exhibit less aggressive fibroblast activity [1]. The fibrotic progression in both scar types involves a dysregulated wound healing response, where inflammatory, epithelial, and stromal signals fail to restore tissue homeostasis after injury, leading to persistent fibroblast activation and ECM accumulation [2]. This aberrant fibrogenesis is orchestrated by multiple cytokines and signaling pathways, with transforming growth factor‐β1 (TGF‐β1) and interleukin‐6 (IL‐6) identified as pivotal pro‐fibrotic mediators. TGF‐β1 promotes fibroblast proliferation, differentiation into myofibroblasts, and excessive ECM production, while IL‐6 plays a crucial role in sustaining inflammatory responses that further drive fibrosis [3, 4, 5, 6, 7, 8]. Clinically, keloids and hypertrophic scars impose significant physical and psychosocial burdens. They often cause pain, pruritus, and restricted mobility depending on location and severity. The psychological impact includes reduced self‐esteem, anxiety, depression, and social stigma, which collectively diminish patients' quality of life [9]. Epidemiologically, these scars disproportionately affect individuals with darker skin phototypes, with higher prevalence in certain ethnic groups, and commonly arise in younger populations, emphasizing the need for effective and targeted intervention [10]. Conventional treatments such as surgical excision, corticosteroid injections, laser therapy, and cryotherapy are frequently limited by suboptimal efficacy, adverse effects, and high recurrence rates, particularly in keloid [11]. Thus, there is an urgent clinical need to develop novel, efficient, and highly specific therapeutic strategies that address the underlying molecular mechanisms of scar fibrosis.

In recent years, targeted inhibition of TGF‐β1 and IL‐6 has become a new strategy for fibrotic diseases' treatment. Studies have shown that using small molecule TGF‐β1 receptor inhibitors can effectively inhibit the transformation of TGF‐β1‐driven fibroblasts into myofibroblasts, thereby reducing collagen synthesis and ECM contraction [7]. In drug delivery systems, alginate‐agarose hydrogels have been widely studied for the treatment of various fibrotic diseases due to their excellent biocompatibility and controlled drug release properties. Research indicates that alginate hydrogels can effectively encapsulate drugs and achieve sustained release in vivo, providing stable drug concentrations and enhancing therapeutic effects [8, 9, 10]. Through computer‐aided design, studying the interaction mechanisms of aptamers with TGF‐β1 and IL‐6 helps us better understand the roles of these factors in keloid and hypertrophic scars formation, thereby providing a theoretical basis for developing new treatment strategies.

This systematic review aims to evaluate and synthesize current evidence to explore the potential of aptamers based on alginate‐agarose hydrogels in inhibiting keloid fibrosis, analyze their interactions with TGF‐β1 and IL‐6, and assess their prospects in clinical applications. By integrating existing literature, we aim to provide new ideas and directions for future research to improve scar treatment outcomes and promote the application of aptamer‐related biomaterials.

## 2 Methods

### 2.1 Literature Search Strategies

A systematic literature search was conducted to identify relevant studies investigating the application of DNA aptamers and alginate–agarose hydrogel‐based delivery systems in the treatment of keloids, hypertrophic scars, and fibrosis‐related conditions. The search was conducted across three electronic databases: PubMed, Web of Science, and Scopus. Publications from January 2000 to March 2025 were considered eligible for inclusion to capture both foundational and recent advances in the field.

The search strategy combined controlled vocabulary terms and free‐text keywords related to pathological scarring, molecular targets, aptamers, and biomaterial‐based drug delivery. Key search terms included combinations of the following: “keloid”, “hypertrophic scar”, “fibrosis”, “DNA aptamer”, “nucleic acid aptamer”, “TGF‐β”, “IL‐6”, “alginate hydrogel”, “agarose”, and “drug delivery system”. Boolean operators (“AND”, “OR”) were used to refine the search strategy. In addition, reference lists of included articles were manually screened to identify potentially relevant studies that were not captured during the initial database search. To accommodate the emerging and heterogeneous nature of this field, we prespecified a stratified evidence framework comprising (A) aptamer‐focused antifibrotic studies, (B) alginate/agarose hydrogel‐focused antifibrotic delivery studies, and (C) integrated aptamer–hydrogel studies.

This stratification was applied during screening and synthesis to improve transparency and reduce selection bias.

### 2.2 Eligibility Criteria

Studies were selected using predefined criteria. Given that aptamer‐based targeting and alginate/agarose hydrogel delivery represent two complementary research streams, eligibility was defined using a stratified inclusion approach. Eligible studies were categorized into one of three prespecified groups:

Group A (Aptamer‐focused evidence):

1. Original research investigating DNA aptamers (selection/design/validation or therapeutic application) targeting fibrosis‐related pathways (e.g., TGF‐β/SMAD axis, IL‐6/IL‐6R signaling, or other profibrotic mediators).
2. Conducted in keloids, hypertrophic scars, or fibrosis‐relevant experimental models (in vitro, ex vivo, or in vivo).
3. Reported at least one fibrosis‐related outcome (e.g., collagen deposition, α‐SMA, ECM remodeling, profibrotic cytokines, fibroblast proliferation/migration, or scar‐related functional outcomes).

Note: alginate/agarose hydrogel delivery was not required for Group A.

Group B (Hydrogel‐focused evidence):

1. Original research investigating alginate‐based and/or agarose‐based hydrogel systems for localized or sustained delivery in keloids, hypertrophic scars, wound scarring, or fibrosis‐relevant models.
2. Reported drug/biologic delivery and at least one fibrosis‐ or scar‐relevant biological/therapeutic outcome (e.g., scar thickness, collagen organization, inflammatory markers, fibroblast response).

Note: aptamer involvement was not required for Group B.

Group C (Integrated aptamer–hydrogel evidence):

1. Original research combining DNA aptamers with alginate/agarose hydrogel‐based delivery in keloid/hypertrophic scar or fibrosis‐relevant models.
2. Reported fibrosis‐related biological/therapeutic outcomes.

General inclusion criteria (all groups):

1. Full‐text accessible, English‐language publications.
2. Published between January 2000 and March 2025.

Exclusion criteria (all groups):

1. Conference abstracts, editorials, commentaries, or letters without original experimental data.
2. Narrative reviews or systematic reviews (used only for background/context, not included in data synthesis).
3. Studies unrelated to pathological scarring/fibrosis or lacking scar/fibrosis‐relevant outcomes.
4. Articles with insufficient methodological detail to support interpretation (e.g., unclear model/assays/intervention), after full‐text review.

### 2.3 Study Selection Process

All retrieved records were imported into reference management software and duplicate entries were removed prior to screening. Titles and abstracts were initially screened for relevance to the scope of this review. Studies that clearly did not meet the inclusion criteria were excluded at this stage.

Full‐text versions of potentially eligible articles were assessed by the authors for final inclusion based on the predefined criteria. Any discrepancies arising during the screening process were resolved through discussion and consensus. The overall study selection process was conducted in a stepwise manner to ensure transparency and reproducibility. During full‐text assessment, eligible studies were assigned to Group A, B, or C according to the prespecified stratified inclusion framework.

### 2.4 Data Extraction and Synthesis

Data were extracted using a standardized framework. Core fields captured across all included studies were publication year, study design, experimental model, intervention/exposure, comparator (if applicable), outcomes, and key findings.

Group‐specific fields were additionally extracted:

Group A: aptamer type and sequence/modifications (if reported), selection/validation method (e.g., SELEX/in silico), molecular target(s), binding/affinity information (if available), and antifibrotic readouts.

Group B: hydrogel composition (alginate/agarose ratio, crosslinking, mechanical properties if available), loading cargo, release kinetics, delivery route, degradation/biocompatibility, and antifibrotic/scar outcomes.

Group C: integration strategy (aptamer immobilization/encapsulation), retention or release behavior, target engagement, and combined biological efficacy endpoints.

Findings were synthesized qualitatively using a stratified thematic synthesis, with results reported separately for Groups A, B, and C and then integrated to identify translational gaps and opportunities for aptamer–hydrogel co‐development.

### 2.5 Assessment of Methodological Limitations and Bias

Considering that most included studies were preclinical in nature, potential sources of bias were evaluated qualitatively. These included variability in experimental models, differences in aptamer sequence design, inconsistencies in delivery systems, and heterogeneity in outcome assessment methods. Limitations related to sample size, short observation periods, and the lack of standardized evaluation criteria were also noted.

Rather than excluding studies based on methodological variability alone, these factors were taken into account during data interpretation to provide a balanced and critical synthesis of the available evidence. This approach was intended to reflect the current developmental stage of aptamer‐based therapies for keloids and hypertrophic scars while highlighting areas requiring further standardization and validation.

### 2.6 Reporting Framework

This review was conducted in accordance with PRISMA 2020 guidelines to enhance transparency and reduce selection bias. Although no formal meta‐analysis was performed, methodological rigor was maintained through predefined search strategies, explicit eligibility criteria, and structured data extraction. The findings are presented in a narrative format aligned with the thematic organization of the included studies. The study selection process will be presented using a PRISMA flow diagram, and included studies will be reported according to the prespecified stratified evidence groups (A–C).

## 3 Results

The study selection process is summarized in Figure 1. The literature search identified a total of 302 records through database searching and citation tracking. Following title and abstract screening, 178 records were retained for further evaluation, while 228 records were excluded as they were considered background studies, clearly irrelevant, or outside the scope of fibrosis‐ or scar‐related outcomes.

**FIGURE 1:**
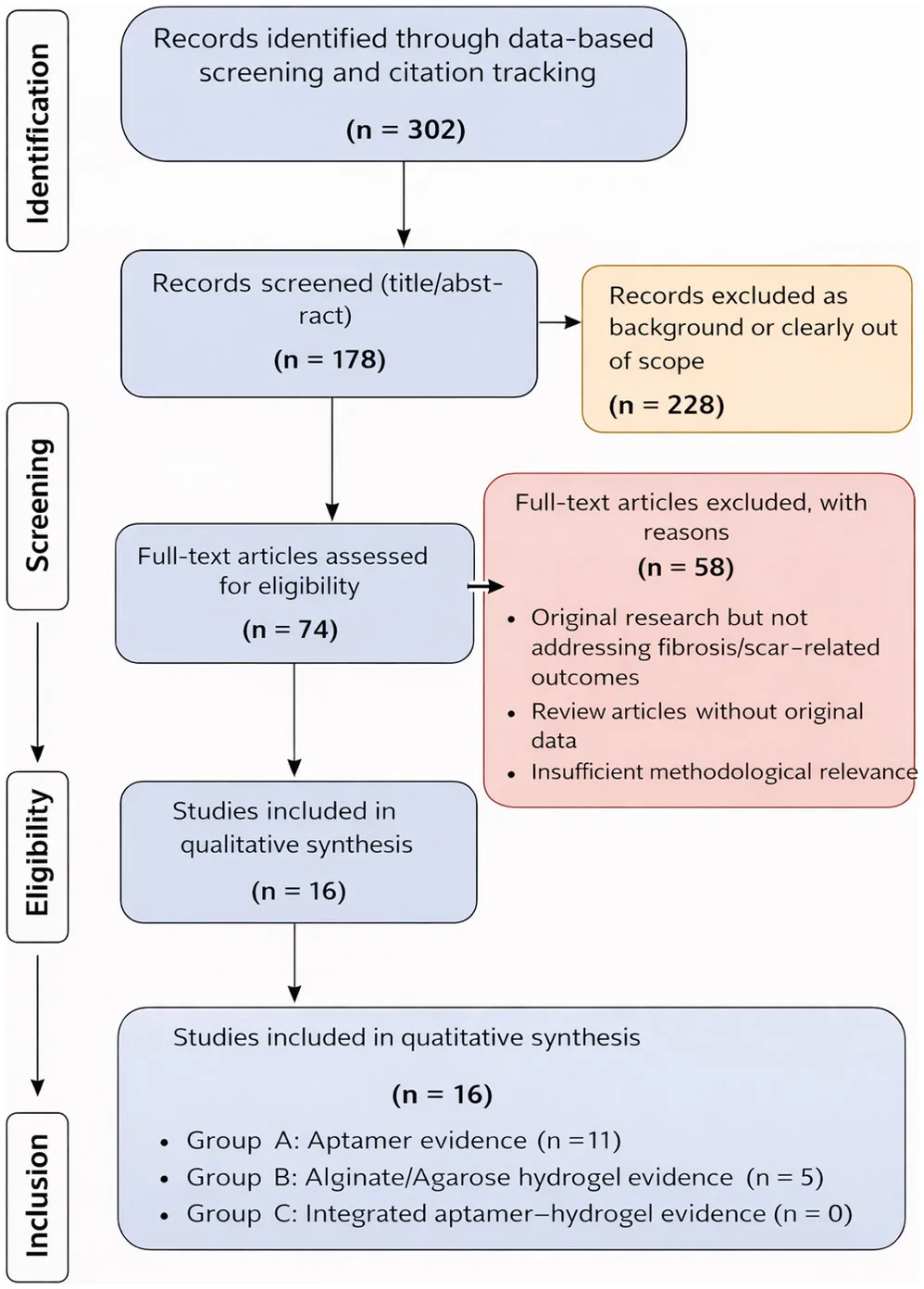
The Preferred Reporting items for systematic reviews (PRISMA) flow diagram is illustrated.

A total of 74 full‐text articles were subsequently assessed for eligibility. Of these, 58 articles were excluded due to the following reasons: (i) lack of relevance to fibrosis or scar‐related outcomes, (ii) review articles without original experimental data, and (iii) insufficient methodological relevance.

Ultimately, 16 studies met the inclusion criteria and were included in the final synthesis. Among these, 11 studies focused on aptamer‐related evidence, 5 studies investigated alginate or agarose hydrogel systems, and no eligible studies were identified that specifically examined integrated aptamer–hydrogel approaches.

### 3.1 Pathological Mechanisms and Current Treatment of Keloids and Hypertrophic Scars

#### 3.1.1 Pathological Characteristics of Keloids and Hypertrophic Scars

Based on the studies included, pathological features of keloids and hypertrophic scars were consistently characterized by excessive extracellular matrix deposition and persistent fibroblast activation. The selected literature collectively demonstrates that aberrant collagen synthesis, altered collagen type ratios, and sustained myofibroblast differentiation represent key histopathological hallmarks underlying pathological scar formation. Keloids and hypertrophic scars are a phenomenon of excessive fibrosis caused by abnormal wound healing processes, with histological features mainly characterized by collagen deposition and sustained activation of fibroblasts. Studies have shown that collagen synthesis is significantly increased in keloid tissue, particularly with an imbalance in the ratio of Type I and Type III collagen, leading to structural changes in the tissue [11, 12]. Fibroblasts in keloids and hypertrophic scars exhibit a highly activated state, with increased proliferation and a tendency to transform into myofibroblasts, accompanied by the upregulation of α‐SMA (smooth muscle alpha‐actin), further promoting excessive collagen synthesis [13]. The infiltration of inflammatory cells, such as macrophages and lymphocytes, can also be observed in scar tissue; these cells promote fibroblast activation and the fibrotic process by releasing various cytokines (such as IL‐6 and TGF‐β1), creating a complex pathological environment as illustrated in Figure 2 [14].

**FIGURE 2:**
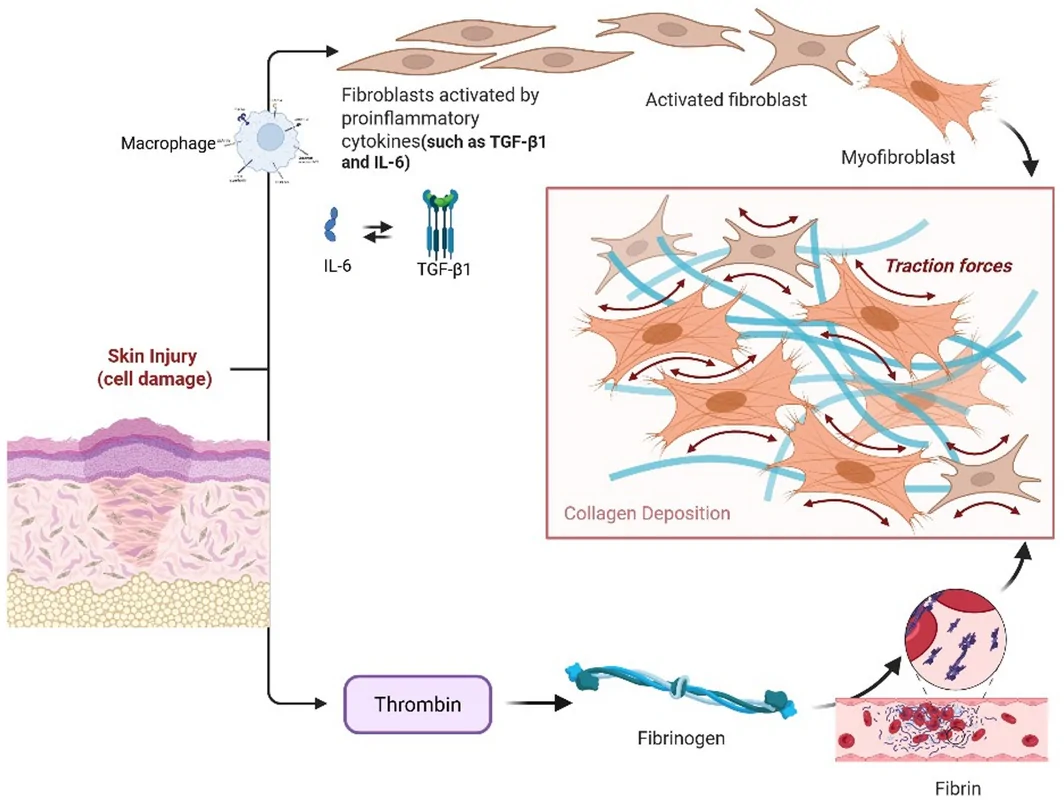
Illustration of keloid and hypertrophic scars pathogenesis is created by BioRender.com. Retrieved from https://app.biorender.com/biorender‐templates.

#### 3.1.2 Role of TGF‐β1 and IL‐6 in Keloids and Hypertrophic Scars

Evidence from the studies included in this review highlights the central involvement of transforming growth factor‐β1 (TGF‐β1) and interleukin‐6 (IL‐6) in the molecular regulation of keloid and hypertrophic scar fibrosis. Across multiple experimental models, these cytokines were repeatedly identified as critical drivers of fibroblast activation, inflammatory persistence, and extracellular matrix accumulation. Transforming Growth Factor‐β1 and Interleukin‐6 are key cytokines in the formation and development of keloids. TGF‐β1 primarily promotes the fibrotic process by activating fibroblasts and enhancing collagen synthesis. In keloids and hypertrophic scars, the expression of TGF‐β1 is significantly elevated, promoting the transformation of fibroblasts into myofibroblasts, which increases collagen matrix accumulation [15]. Additionally, IL‐6, as an important pro‐inflammatory cytokine, can enhance the fibrotic response through the STAT3 signaling pathway, causing fibroblasts to remain activated in an inflammatory environment, thereby exacerbating scar formation [16]. Studies have shown that inhibiting the signaling pathways of TGF‐β1 or IL‐6 can effectively slow down keloid and hypertrophic scar formation, suggesting that these factors may serve as potential therapeutic targets [17].

#### 3.1.3 Limitations of Current Treatment Methods

The reviewed studies evaluating existing therapeutic approaches for keloids and hypertrophic scars consistently report suboptimal long‐term outcomes and high recurrence rates. Collectively, the evidence underscores the limited ability of conventional interventions to adequately modulate key profibrotic signaling pathways or provide sustained, targeted antifibrotic effects. Currently, treatment methods for keloids and hypertrophic scars primarily include surgical excision, steroid injections, and local radiotherapy (Table 1). However, conventional treatments address the physical manifestations but fail to consistently modulate the underlying fibrotic processes or prevent recurrence. These traditional treatment methods have certain limitations [18, 19, 20, 21, 22]. For instance, while surgical excision can remove prominent keloids, it often leads to the formation of new scars and has a longer recovery period [21]. Steroid injections help reduce local inflammation and inhibit collagen synthesis, but their effects are often limited and may have side effects [23]. Additionally, while radiotherapy can effectively reduce scar recurrence in some cases, the potential risks of radiation damage limit its application [24, 25]. Central profibrotic cytokines including transforming growth factor‐beta (TGF‐β), interleukin‐6 (IL‐6), and platelet‐derived growth factor (PDGF) play dominant roles in driving fibroblast activation, ECM deposition, and scar progression [26, 27]; however, existing therapies do not specifically inhibit these pathways with sufficient efficacy or selectivity. Moreover, drug delivery systems used in scar management often lack sustained release and targeted delivery capabilities, limiting therapeutic concentrations at the fibrotic site and increasing systemic side effects [28]. The absence of delivery platforms that can provide controlled, localized, and prolonged release of anti‐fibrotic agents reduces treatment effectiveness and patient compliance. Emerging strategies employing nanomedicine, cell‐based therapies, and molecularly targeted agents show promise but require further development to overcome challenges related to stability, targeting, and safety [27, 28]. Therefore, there is a critical need for advanced therapeutic systems integrating molecular targeting of key profibrotic factors with optimized delivery mechanisms to enhance treatment outcomes in keloids and hypertrophic scars [29].

**TABLE 1:** Current treatment methods in clinic for keloids and hypertrophic scars.

Treatments	Mechanism of action	Clinical efficacy	Advantages	Potential side effects
Silicone dressing	Create an occlusive barrier, maintain hydration of stratum corneum, reduce collagen production	Effective in reducing scar height, redness, and pruritus when used consistently	Non‐invasive, easy to use, transparent, can be worn discreetly, suitable for various body parts	Minimal side effects, occasional skin irritation or maceration
Topical corticosteroids	Penetrate skin, interact with glucocorticoid receptors, reduce production of inflammatory mediators	Effective in flattening keloid scars and reducing symptoms when used in conjunction with other therapies	Non‐invasive, suitable for patients not candidates for aggressive treatments	Long‐term use can lead to skin atrophy, telangiectasia; requires careful monitoring
Cryotherapy	Freezes scar tissue, causing cell injury and ischemic necrosis, reducing keloid volume and symptoms	Reduces keloid scar volume by an average of 51%–63%; scar recurrence rates range from 0% to 24%	Non‐invasive, can be combined with other treatments	Hypopigmentation, particularly in darker skin types
Surgical options	Remove keloid tissue, often combined with adjuvant therapies to minimize recurrence	Effective for large or resistant keloids; recurrence rates vary	Directly removes bulk of keloid, improves cosmetic appearance	High recurrence rates if not combined with adjuvant therapies; risk of infection, scarring
Radiation therapy	Inhibits fibroblast proliferation and collagen production, usually administered post‐surgery	Reduces recurrence rates significantly when used as adjuvant to surgery	Non‐invasive, effective in combination with surgery	Temporary erythema, hyperpigmentation; rare long‐term effects: skin atrophy, telangiectasia
Laser therapies	Target scar tissue with specific wavelengths of light, reduce size, color, and symptoms	Effective in improving appearance and reducing symptoms, especially when combined with other treatments	Non‐invasive to minimally invasive, can be tailored to scar characteristics	Temporary redness, swelling, changes in pigmentation; requires multiple sessions
Intralesional injections	Deliver therapeutic agents directly into keloid tissue to reduce size and symptoms	Effective in flattening scars and alleviating symptoms; varies by agent	Good response rates ranging from 50% to 90% when combined with other treatments, such as surgery, 5‐fluorouracil, and cryotherapy	Skin atrophy, pain, hypopigmentation, telangiectasia with repeated corticosteroid/Fluorouracil/Bleomycin/Botulinum toxin injections
Pressure garment therapy	Reduce perfusion and oxygen concentrations reaching the scar surface resulting in reduced collagen synthesis	Effective in reducing hydration, decreases angiogenesis, and induces apoptosis	Help to reduce pain and itching associated with scars	Inconvenience caused by the garments and its costs significantly decrease its compliance
Photodynamic therapy (PDT)	PDT reduces fibroblast proliferation which inhibits collagen synthesis	Topical photodynamic therapy (PDT) had been used in the treatment of a number of dermatologic conditions (basal cell carcinoma, Bowen's disease, and actinic keratosis) and had been suggested as a therapeutic modality for scars	Qualify as a promising non‐invasive modality for treating excessive scarring and improving their cosmetic outcome with minimal side effects	With a limited number of data available
Cryotherapy	Microvascular damage and ischemic necrosis of scars are the main modes of acting cryotherapy	Currently, used protocols of treatment administer cryotherapy sessions every 2–3 weeks to guarantee good post‐operative healing	Limit side effects such as hypopigmentation, skin atrophy, and pain	Combination with other treatment modalities and at early stages of scare formation
Percutaneous collagen induction with microneedling	Puncturing the skin with microneedles results in controlled zones of microinjury to the papillary dermis which triggers new collagen and elastin formation to replace and remodulate old scar	Microneedling is a non‐invasive procedure that uses needles to create controlled skin injury	Assisted drug delivery had been shown to improve drug delivery and enhance cosmetic outcome of scars	Requires more validation by future large scale studies
Fat grafting	Autologous fat grafting stem cells (ADSCs) inhibite fibroblast proliferation and ECM formation	The procedure causes less fibrosis, pain re duction, and more flexibility in areas of scar contraction	Improve scar texture, pliability volume, and elasticity	Requires more validation by future large scale studies

### 3.2 Design and Screening of DNA Aptamers

The included studies investigating DNA aptamer development predominantly focused on improving binding specificity and affinity toward fibrosis‐associated molecular targets. These studies employed a range of in silico and experimental screening strategies to identify aptamers with potential relevance for antifibrotic applications.

#### 3.2.1 Computer‐Aided Design of High‐Affinity Aptamers

Across the included literature, computer‐aided design approaches were commonly applied to facilitate the identification and optimization of high‐affinity DNA aptamers. Molecular docking, structural modeling, and kinetic simulations were frequently used to predict aptamer–target interactions prior to experimental validation. The application of computer‐aided design technology in the design of DNA aptamers significantly enhances the screening efficiency and specificity of aptamers (Figure 3). Molecular docking technology can simulate the interactions between aptamers and target molecules, predicting their binding capabilities. This technology helps researchers select high‐affinity aptamers by calculating the binding energy between aptamers and target molecules (such as proteins or small molecules). For example, some studies have utilized molecular docking methods to design DNA aptamers targeting specific biomarkers to improve their binding specificity and stability [30]. Moreover, kinetic simulation techniques can be used to assess the dynamic changes during the binding process of aptamers with target molecules, analyzing their binding and dissociation rates, which is crucial for optimizing aptamer design. By combining these two technologies, researchers can quickly generate and screen DNA aptamers with desirable binding characteristics in silico, providing a reliable theoretical basis for subsequent experiments [22, 26].

**FIGURE 3:**
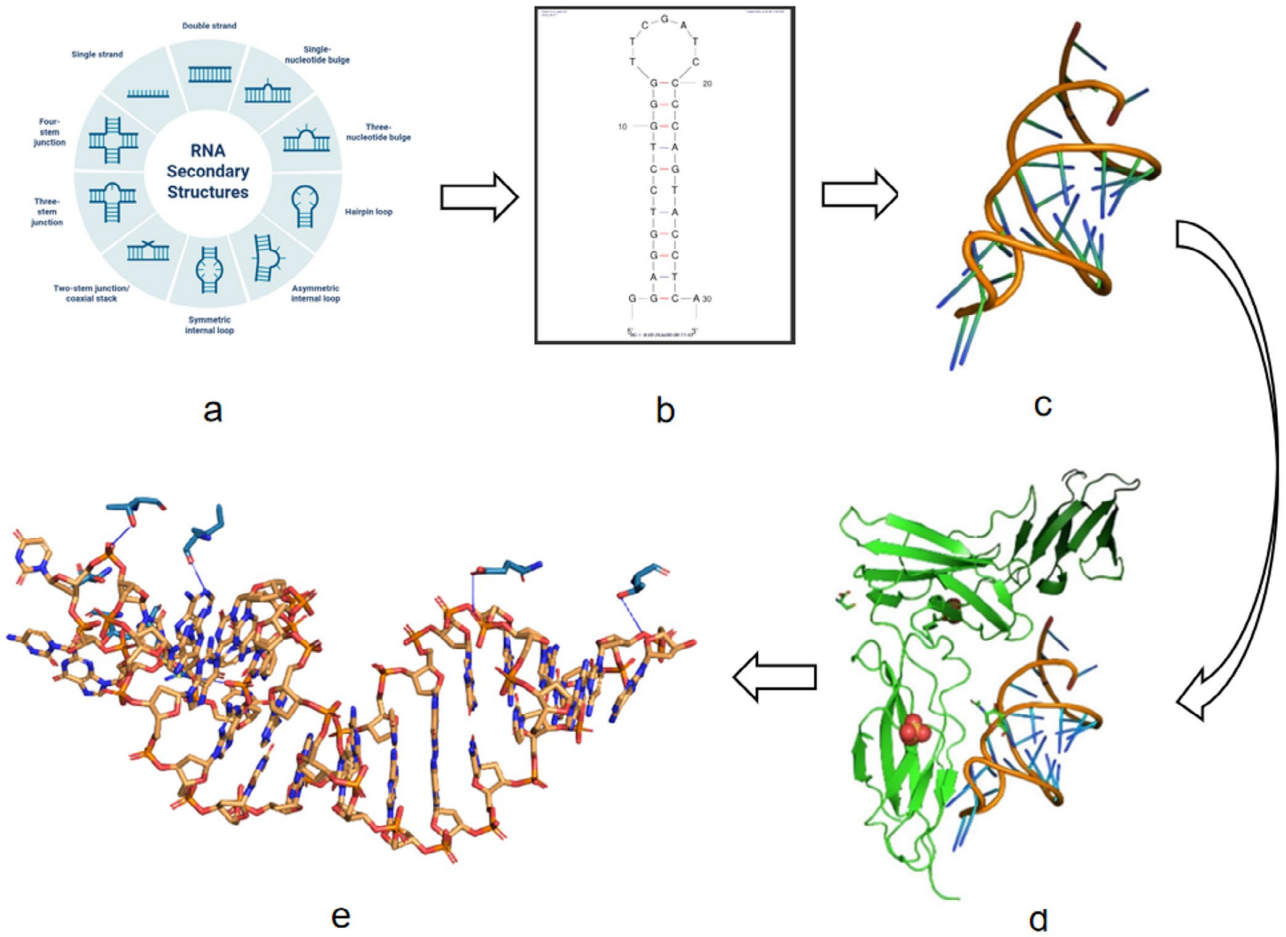
Procedures of target protein combine with selected specific aptamer in silico Computer. (a) Prediction of RNA Stuctures; (b) Selection of efficient and specific aptamer in 2D; (c) Selected aptamer in 3D; (d) Molecular docking technology; (e) High‐affinity aptamer by calculating the binding energy between aptamer and target molecule.

#### 3.2.2 Binding Characteristics of Aptamers With TGF‐β1/IL‐6

Studies included in this review consistently examined the interaction profiles between DNA aptamers and profibrotic cytokines, particularly TGF‐β1 and IL‐6. These investigations provided qualitative evidence regarding binding affinity, specificity, and downstream signaling modulation. Aptamers are emerging molecular recognition tools with high specificity and affinity toward target proteins, offering considerable potential in targeted therapeutic applications. The binding characteristics between aptamers and profibrotic cytokines, such as transforming growth factor‐β1 (TGF‐β1) and interleukin‐6 (IL‐6), are critical parameters for evaluating their translational relevance in fibrotic diseases. Biophysical techniques, including Surface Plasmon Resonance (SPR) and Isothermal Titration Calorimetry (ITC), are commonly employed to quantify aptamer–target interactions, providing precise binding affinity constants and insights into binding site characteristics [31].

Current studies investigating TGF‐β1‐ and IL‐6‐targeting aptamers are largely based on in silico design and validation in oncologic or generalized fibrotic models, as summarized in Table 2. To date, aptamers specifically developed and validated for keloid‐derived fibroblasts or keloid tissue remain limited, highlighting an important gap in the current literature. Nevertheless, these oncologic and fibrotic tumor models share key profibrotic signaling pathways with keloid pathogenesis, particularly the aberrant activation of TGF‐β1 and IL‐6 signaling cascades, which are central drivers of fibroblast activation and extracellular matrix deposition [35].

**TABLE 2:** Summary of included studies on aptamer and hydrogel‐based strategies for keloid fibrosis inhibition.

Author (year)	Study type	Model	Intervention	Hydrogel	Outcomes	Mechanism
Abune et al. 2022	Aptamer‐based	In vitro/in vivo	Targeted aptamer delivery	Hydrogel (unspecified)	ECM deposition; controlled drug release	ECM remodeling
Ando et al. 2021 [32]	Aptamer‐based	In vitro	Aptamer targeting IL‐6	Not reported	Reduced IL‐6 levels	IL‐6 pathway inhibition
Chen et al. 2011 [33]	Aptamer‐based	In vitro	Anti‐inflammatory aptamer	Not reported	Decreased inflammatory markers	IL‐6 pathway modulation
Gupta et al. 2014	Aptamer‐based	In vitro	IL‐6‐targeting aptamer	Not reported	Reduced fibroblast proliferation and inflammation	IL‐6 signaling; inflammation suppression
Kim et al. 1995 [34]	Aptamer‐based	In vitro	Cytokine‐targeting aptamer	Not reported	Reduced IL‐6 expression	IL‐6 pathway inhibition
Li et al. 2025	Hydrogel‐based	In vitro	Alginate hydrogel delivery	Alginate	Enhanced drug release; reduced fibroblast proliferation	Not reported
Lo et al. 2022	Aptamer‐based	In vitro	Targeted aptamer delivery	Not reported	Reduced fibroblast migration; improved delivery	Not reported
Meng et al. 2023	Hydrogel‐based	In vitro	Alginate scaffold	Alginate	Reduced fibroblast proliferation	Not reported
Meyer et al. 2014	Aptamer‐based	In vitro	IL‐6‐targeting aptamer	Not reported	Reduced inflammatory response	IL‐6 signaling modulation
Meyer et al. 2012	Aptamer‐based	In vitro	Aptamer‐based targeting	Not reported	Decreased IL‐6 expression	IL‐6 pathway inhibition
Nakamura et al. 2021	Aptamer‐based	In vivo	Targeted molecular therapy	Not reported	Reduced scar thickness; improved wound closure	Not reported
Sahoo et al. 2021	Hydrogel‐based	In vivo	Alginate hydrogel system	Alginate	Improved wound healing; sustained release; biocompatibility	Not reported
Singh et al. 2022	Hydrogel‐based	In vitro/in vivo	Alginate biomaterial	Alginate	Reduced collagen deposition; good biocompatibility	ECM remodeling
Sánchez‐Salcedo et al. 2023	Aptamer‐based	In vitro	Anti‐inflammatory aptamer	Not reported	Reduced IL‐6 levels	IL‐6 pathway inhibition
Takahashi et al. 2022	Aptamer‐based	In vitro	TGF‐β‐targeting aptamer	Not reported	Inhibited TGF‐β signaling	TGF‐β/SMAD pathway suppression
Zeng et al. 2015	Hydrogel‐based	In vivo	Composite hydrogel delivery	Alginate–agarose	Improved wound healing; controlled release	Not reported
Zhu et al. 2012	Aptamer‐based	In vitro	Anti‐fibrotic aptamer	Not reported	Reduced collagen, α‐SMA expression	TGF‐β/SMAD inhibition; fibroblast activation suppression

Abbreviations: ECM, extracellular matrix; IL‐6, interleukin‐6; TGF‐β, transforming growth factor beta; α‐SMA, alpha‐smooth muscle actin.

Despite these shared molecular pathways, notable differences exist between keloids and fibrotic tumors, including cellular composition, extracellular matrix architecture, mechanical microenvironment, and inflammatory context. These distinctions underscore the necessity of developing keloid‐specific aptamers through targeted selection strategies and disease‐relevant experimental models. Therefore, existing TGF‐β1‐ and IL‐6‐targeting aptamers should be viewed as proof‐of‐concept candidates rather than direct therapeutic solutions for keloid treatment.

In summary, while current evidence supports the feasibility of targeting TGF‐β1 and IL‐6 using aptamers, further studies are required to establish keloid‐specific aptamer designs and to elucidate their molecular mechanisms within scar‐specific microenvironments. Such efforts are essential for advancing aptamer‐based therapeutic strategies toward clinical application in keloid management.

### 3.3 Synthesis and Optimization of Aptamer‐Conjugated Alginate‐Agarose Hydrogels

The reviewed studies exploring aptamer‐conjugated alginate–agarose hydrogels primarily addressed material synthesis, structural stability, and delivery performance. Collectively, these studies aimed to optimize hydrogel formulations to support sustained and localized release of bioactive molecules.

#### 3.3.1 Cross‐Linking Strategies for Structural Stability

Dynamic borate ester/diol bonds between alginate and borax enhance hydrogel mechanical properties and self‐healing [27]. Homogeneous calcium phosphate‐alginate hybrids enable injectable, tunable hydrogels with cell‐compatible porous structures [28].

The structural stability of alginate‐agarose hydrogels critically depends on optimized crosslinking strategies [22]. Studies demonstrate that borax, a natural polysaccharide crosslinker, forms dynamic and reversible boronate/diol bonds with cis‐diol‐containing polysaccharides (e.g., alginate), significantly enhancing mechanical strength and self‐healing capacity. This unique crosslinking mechanism not only improves hydrogel stability but also confers responsiveness to temperature, pH, and ionic conditions, broadening its biomedical and tissue engineering applications [27].

Additionally, hydroxyapatite‐alginate hybrids (CaP‐alginate) act as uniformly dispersed calcium sources, enabling homogeneous gelation to produce injectable hydrogels with tunable mechanical properties [28]. These crosslinking approaches provide a robust foundation for constructing stable aptamer‐hydrogel composites, advancing their therapeutic potential [29].

#### 3.3.2 Controlled Release Kinetics of Aptamers

The controlled‐release kinetics of aptamers in alginate‐agarose hydrogels play a pivotal role in optimizing their therapeutics. Studies demonstrate that aptamers can bind to hydrogels via encapsulation during gelation or incorporation into pre‐formed hydrogels, exhibiting specific affinity for alginate [29]. For instance, aptamer‐bovine serum albumin (BSA) conjugates significantly reduce diffusion rates within hydrogels, enabling sustained release over 1 week [36]. Furthermore, incorporating vascular endothelial growth factor (VEGF)‐targeting aptamers decreases the apparent diffusion coefficient of VEGF by two orders of magnitude, prolonging retention and release duration [31]. These findings highlight the capacity of functionalized aptamer designs to precisely regulate biomolecule release kinetics, offering a versatile strategy for targeted therapeutic delivery.

#### 3.3.3 Mechanical Properties and Degradation Profiles

The included literature assessing mechanical performance and degradation characteristics demonstrates that alginate–agarose hydrogels can be tailored to meet the biomechanical requirements of fibrotic tissue environments. Variations in composition and fabrication conditions were shown to directly affect scaffold durability and degradation behavior. Alginate/hydroxyapatite hybrids demonstrate injectability, tunable mechanics, and cytocompatibility, guiding hydrogel‐aptamer composite design [37].

The mechanical properties and degradation characteristics of alginate‐agarose hydrogels critically influence their clinical performance [35]. In practical applications, these parameters determine whether the hydrogel can function effectively as a drug carrier or tissue scaffold [38, 39]. Optimizing the pH and ratio of alginate to chitosan enables the fabrication of microspheres with homogeneous composite structures, where precise control of fabrication parameters ensures structural uniformity [40]. Surface charge, hydrophilicity, and roughness of these microspheres can be finely tuned, expanding their utility across diverse applications [37, 41].

This homogeneous composite hydrogel exhibits remarkable properties, including superior swelling capacity and wettability, which enhance the viability and functionality of encapsulated hepatocytes [42].

Furthermore, alginate/hydroxyapatite hybrid hydrogels possess uniform porous architectures. Parameters such as gelation time, injectability, and mechanical strength can be optimized by adjusting fabrication conditions, resulting in excellent cytocompatibility and broad biomedical potential [28]. The porous structure supports cell proliferation and maintains physiological functions, making it ideal for regenerative medicine [43].

These studies provide critical insights into the mechanical and degradation behaviors of aptamer‐hydrogel composites, advancing both theoretical knowledge and practical applications in biomedicine.

### 3.4 In Vitro Evaluation of Aptamer‐Hydrogel Complexes

In vitro studies included in this review primarily focused on evaluating the structural stability, release behavior, and biological performance of aptamer–hydrogel complexes under physiologically relevant conditions.

#### 3.4.1 Stability Testing of Complexes

The reviewed in vitro investigations assessed hydrogel stability under diverse environmental conditions, providing evidence for the structural robustness and functional reliability of alginate‐based delivery systems. In the development of aptamer‐hydrogel complexes, assessing their structural integrity in different environments is crucial to ensure their effectiveness and safety in clinical applications. Studies have shown that alginate‐based hydrogels, due to their excellent biocompatibility and adjustable mechanical properties, have become the foundation for various biomedical materials. For example, one study explored the application of alginate hydrogels in preventing peritoneal adhesions, and the results showed that the hydrogels could effectively promote the proliferation and migration of human peritoneal mesothelial cells while inhibiting the production of transforming growth factor beta 1 (TGF‐β1), thus preventing the occurrence of peritoneal fibrosis [44]. Furthermore, the degree of cross‐linking of alginate hydrogels and environmental conditions (such as pH and ionic strength) directly affect their stability and drug release characteristics [45, 46, 47]. By adjusting these parameters, the performance of hydrogels can be optimized to adapt to various biological environments.

In stability testing, common methods include immersion tests and dynamic mechanical analysis to evaluate the physical and chemical stability of hydrogels in vitro [39, 48]. Specifically, by immersing hydrogel samples in different physiological environments (such as varying ionic concentrations and pH values) and monitoring changes in mass and mechanical performance degradation, long‐term usability can be effectively assessed [49]. Additionally, techniques such as scanning electron microscopy (SEM) can be employed to observe microscopic structural changes in hydrogels, providing intuitive evidence for their stability. Research has shown that mixed hydrogels of alginate and silk fibroin exhibit excellent structural stability and biocompatibility in these tests, indicating their potential for future biomedical applications [48, 50].

#### 3.4.2 Drug Release Kinetics

Across multiple experimental studies, release kinetics were systematically analyzed to characterize the temporal profile of aptamer diffusion from hydrogel matrices and to predict therapeutic availability. The release behavior of aptamers and their influencing factors is crucial in studying aptamer‐hydrogel complexes. Drug release kinetics not only affect therapeutic efficacy but also relate to the design and preparation of hydrogels. Studies have shown that the release characteristics of aptamers are influenced by various factors, including the cross‐linking density of hydrogels, the molecular weight of aptamers, and external environments such as temperature, pH, and ionic strength. For example, one study found that platelet‐rich plasma (PRP) embedded in alginate hydrogels exhibited rapid initial release in vitro, followed by a sustained release characteristic, which helps improve muscle injury repair while reducing fibrosis [51].

In the study of drug release kinetics, in vitro release experiments are typically conducted by placing hydrogel samples in physiological saline or simulated body fluids, with regular sampling and analysis of the released amount of aptamers to evaluate their release efficiency. Common mathematical models (such as zero‐order release, first‐order release, etc.) can help describe the release process of aptamers, thus providing a theoretical basis for further research and clinical applications. Various release models (zero‐order release model, first‐order release model, and Higuchi release model) can assist researchers in understanding and predicting the release characteristics of aptamers. Moreover, studies have emphasized the influence of the physical properties of hydrogels (such as pore size and surface hydrophobicity) on the release rate of aptamers, which directly affects the bioavailability and therapeutic efficacy of drugs in vivo [52]. By optimizing these parameters, precise control over aptamer release will be achieved to some extent, laying the foundation for subsequent clinical applications.

### 3.5 Biocompatibility Study of Aptamers on Fibroblasts

The studies included in this review consistently evaluated the biocompatibility of aptamers using fibroblast‐based assays, with a focus on cytotoxicity, proliferation, and apoptosis regulation.

#### 3.5.1 Cytotoxicity Test

Cytotoxicity assessments reported in the included studies demonstrated that aptamers exhibit minimal adverse effects on fibroblast viability within defined concentration ranges. Aptamers, as emerging biomaterials, exhibit good biocompatibility, and their cytotoxicity assessment on human keloid dermal fibroblasts (HKDFs) is key to studying their application in keloid treatment. Cytotoxicity tests typically employ the MTT assay to determine cell viability. The MTT assay evaluates cellular response to drugs or materials by measuring cellular metabolic activity [53]. In one study, researchers treated cells with aptamers at different concentrations, and the results showed that within a certain concentration range, aptamers exhibited very low cytotoxicity to HKDFs, with cell viability exceeding 90% [54]. Additionally, further experimental results indicated that the biocompatibility of aptamers is reflected not only in low cytotoxicity but also in their support for cell functions, such as promoting cell adhesion and proliferation [55]. These results provide a solid foundation for the use of aptamers in the treatment of keloids and hypertrophic scars, demonstrating their potential as a biomaterial.

#### 3.5.2 Cell Proliferation and Apoptosis

Evidence from cellular studies suggests that aptamers modulate fibroblast proliferation and apoptosis in a context‐dependent manner, potentially contributing to the restoration of fibrotic homeostasis. The investigation into the impact of the aptamer on the proliferation and apoptosis of fibroblasts is a crucial component in comprehending its mechanism of action in the treatment of keloids and hypertrophic scars. In experiments focused on cell proliferation, the application of the aptamers may modulate fibroblast proliferation in a context‐dependent manner. Studies show that the aptamer promotes the progression of the cell cycle by activating intracellular signaling pathways, leading to an increase in cell numbers in cancer [56]. In apoptosis analysis, the application of the aptamer demonstrates potential to inhibit cell apoptosis. Through techniques such as flow cytometry and Western blot, researchers found that the aptamer can downregulate the expression of apoptosis‐related proteins, such as the significant changes in the ratio of Caspase‐3 to Bcl‐2, suggesting that the aptamer may enhance the survival ability of fibroblasts by inhibiting apoptosis. This inhibitory effect on cell death is particularly vital for the treatment of keloids, given that excessive proliferation and an imbalance of apoptosis in fibroblasts can lead to increased fibrosis. Overall, the aptamer not only effectively regulates the proliferation of fibroblasts but also significantly suppresses cell apoptosis. This dual effect provides a new strategy and idea for the treatment of keloids. Future research needs to further explore the specific mechanisms of the aptamer in cell signaling pathways to achieve more precise clinical applications.

### 3.6 Immune Response and Safety Evaluation

Safety‐related outcomes reported in the included studies primarily addressed immunogenicity and long‐term biocompatibility of alginate–agarose‐based aptamer delivery systems.

#### 3.6.1 Immunogenicity Testing

The reviewed literature indicates that alginate‐based hydrogels generally exhibit low immunogenicity, with minimal inflammatory responses observed in preclinical models. In studying the alginate‐agarose hydrogel‐based aptamer, assessing its immune response is a crucial step. Immunogenicity testing typically includes evaluating the immune response of the aptamer and hydrogel to determine whether it may cause adverse immune reactions. According to existing studies, alginate, as a natural polymer material, has good biocompatibility and can effectively reduce the incidence of immune reactions [57]. For example, some studies indicate that alginate hydrogel does not cause significant immune reactions when used in vivo and can promote cell proliferation and migration, thus aiding tissue repair and regeneration [58, 59]. Additionally, research has shown that alginate hydrogel has significant effects in promoting angiogenesis and inhibiting fibrosis [60], which may be related to its low immunogenicity. These results suggest that selecting appropriate polymer materials can effectively reduce immunogenicity when designing new hydrogels, thereby improving their performance in immunogenicity testing. Generally, immunogenicity testing requires systematic evaluation using small animal models. By observing immune responses (such as antibody production, cytokine release, etc.) in animals after injection, researchers can better understand the safety and efficacy of the hydrogel. For instance, in one study, an alginate‐agarose composite was used to release growth factors without causing significant inflammatory responses, indicating its safety for in vivo applications [61]. This assessment not only helps to understand the biocompatibility of materials but also provides important theoretical support for subsequent clinical applications.

#### 3.6.2 Long‐Term Safety

Long‐term evaluations reported in the included studies assessed hydrogel degradation behavior and tissue responses, providing preliminary evidence for sustained biocompatibility. Analyzing the long‐term biocompatibility of the composite in vivo is an important aspect of ensuring the success of its clinical application. Long‐term safety evaluation typically involves systematic research on the material's degradation, absorption, and potential chronic inflammatory responses in vivo. It is noteworthy that the degradation rate of alginate‐agarose hydrogel in vivo and the biocompatibility of its degradation products are key factors in evaluating its long‐term safety. Studies have shown that the degradation process of alginate hydrogel is mild, and its degradation products are usually non‐toxic, contributing to tissue regeneration. The degradation process does not induce significant inflammatory responses and has minimal impact on surrounding tissues, indicating its long‐term safety for use. Furthermore, as the hydrogel degrades, the bioactive substances released can further promote cell proliferation and tissue repair, forming beneficial biological effects [62].

Long‐term safety evaluation should also focus on the mechanical properties of the hydrogel and its biomechanical effects on surrounding tissues. Long‐term observations conducted in animal models have shown that the application of the alginate‐agarose composite in vivo can effectively reduce the occurrence of fibrosis and promote tissue regeneration, thereby enhancing its safety and efficacy in clinical applications [63, 64]. Through these evaluations, important theoretical foundations and practical experiences can be provided for future clinical trials.

## 4 Discussion

### 4.1 Future Research Directions and Challenges

Based on the collective findings of the included studies, several research gaps and translational challenges were identified, particularly regarding keloid‐specific aptamer design and clinical applicability.

#### 4.1.1 Aptamer Optimization

Optimizing the aptamer is a crucial part of current research, especially for the treatment of keloids and hypertrophic scars. Enhancing the stability and affinity of the aptamer is key to achieving its clinical application. Research shows that the stability of aptamers is influenced by various factors, including their chemical structure, synthesis methods, and environmental conditions. By introducing non‐natural nucleotides or improving synthesis techniques, the thermal stability and enzymatic resistance of the aptamer can be significantly enhanced, thereby prolonging its half‐life in vivo [65, 66]. Additionally, optimizing the binding affinity of the aptamer to target molecules is also an important direction. Using computer‐aided drug design (CADD) and high‐throughput screening technologies can quickly identify variants of aptamers with stronger affinities. Recent studies have indicated that aptamers developed using these new technologies exhibit superior specificity and affinity when binding to Transforming Growth Factor β (TGF‐β), effectively inhibiting the fibrosis process [67, 68]. Therefore, future research should focus on combining advanced nanotechnology and materials science to improve the biocompatibility and targeting of aptamers, ensuring their safety and efficacy in vivo.

#### 4.1.2 Challenges in Clinical Translation

Despite encouraging advances at the experimental level, multiple obstacles continue to hinder the clinical translation of aptamer‐based therapies for keloid management. One major limitation lies in the requirement for large‐scale manufacturing processes that comply with Good Manufacturing Practice (GMP) standards. The production of chemically modified aptamers and their delivery platforms often entail considerable technical demands and costs, which may restrict clinical scalability.

Another significant challenge concerns the evaluation of safety and efficacy in human populations. Keloids display pronounced clinical and biological heterogeneity, influenced by factors such as anatomical site, genetic predisposition, and recurrence tendency. Clinical evidence suggests that this variability can lead to differential therapeutic responses among patients, complicating trial design and outcome interpretation for antifibrotic agents, including aptamers.

In addition, although aptamers are generally regarded as having low immunogenic potential compared with protein‐based biologics, the possibility of immune recognition following repeated administration cannot be entirely excluded. This issue is particularly relevant in keloid therapy, which often requires prolonged or repeated treatment to control disease progression.

Furthermore, the high rate of keloid recurrence following conventional interventions highlights the need for novel therapeutic paradigms. Rather than being applied in isolation, keloid‐specific aptamers may hold greater clinical value as components of combination treatment strategies, such as adjunctive therapy following surgical excision or in conjunction with intralesional corticosteroids. Addressing these translational challenges through optimized molecular design and tailored clinical strategies will be essential for improving the long‐term effectiveness of aptamer‐based therapies in keloid patients.

#### 4.1.3 Limitations of the Review

Despite adherence to a structured screening framework, several limitations of this systematic review should be acknowledged. First, the review was conducted using a reference‐based systematic approach rather than a fully database‐driven search strategy. Although this method was deliberately selected to capture highly relevant studies dispersed across interdisciplinary domains—including biomaterials engineering, nucleic acid therapeutics, and fibrosis research—it may have resulted in the omission of eligible studies not identified through reference list screening or citation tracking.

Second, the overall number of included studies remains limited, particularly for integrated aptamer–hydrogel strategies (Group C). Most available evidence is derived from preclinical investigations using heterogeneous experimental models, which restrict cross‐study comparability and precludes quantitative meta‐analysis. As a result, the present synthesis is necessarily qualitative and interpretive in nature.

Third, substantial variability exists among the included studies in terms of aptamer design, hydrogel composition, delivery routes, and outcome measures. The lack of standardized experimental protocols and uniform fibrosis‐specific endpoints introduces uncertainty when extrapolating findings across models or toward clinical applications.

Finally, clinical evidence directly evaluating aptamer‐based interventions in keloid patients is currently scarce. Consequently, the translational relevance of many experimental findings remains indirect and should be interpreted with caution. Future updates incorporating database‐based systematic searches and emerging clinical data will be essential to validate and extend the conclusions presented herein.

## 5 Conclusion

This review provides an overview of emerging strategies that aim to regulate fibrotic processes through molecular targeting and controlled delivery. The analyzed studies indicate that aptamers can interact with specific mediators involved in fibrosis, particularly those associated with TGF‐β and IL‐6 signaling, thereby influencing key cellular events such as fibroblast activation and matrix production.

In parallel, hydrogel‐based delivery systems have been investigated for their capacity to localize therapeutic agents within fibrotic tissues. Materials such as alginate and agarose offer structural and physicochemical properties that support sustained release and improved tissue retention, which may enhance therapeutic effectiveness in localized fibrosis.

A notable limitation across the included studies is the predominance of preclinical models and the diversity in experimental approaches. Differences in model selection, intervention design, and outcome assessment reduce comparability and complicate interpretation of overall efficacy.

Another important finding is the scarcity of studies that combine aptamer‐based targeting with hydrogel delivery systems. While both strategies independently show promise, their integration remains largely unexplored, suggesting a potential direction for future research.

From a translational standpoint, several challenges need to be addressed, including improving aptamer stability, optimizing hydrogel formulations for clinical application, and generating evidence from more clinically relevant models. Addressing these issues will be essential for moving these approaches toward therapeutic use.

Overall, advancing this field will require coordinated efforts to integrate targeting precision with delivery efficiency, as well as the establishment of standardized evaluation frameworks.

## Author Contributions

**Xiaorui Hu:** writing – review and editing, writing – original draft, visualization, validation, methodology, formal analysis, data curation, conceptualization. **Nur Azida Mohd Nasir, Shazana Hilda Shamsuddin**, and **Nur Fatiha Ghazalli** have the same contributions as my cosupervisors: writing – review and editing, visualization, validation, methodology, formal analysis, data curation. **Nurul Syazana Mohamad Shah:** writing – review and editing, conceptualization, methodology, data analysis.

## Funding

This work was supported by the Ningxia Province Science and Technology Foundation–Autonomous Region Key R&D Program of China (Grant No. 2025FRG05018) and Medical Research Grant (GPP) Grant No. #2025/769/GPP40.

## Ethics Statement

The authors have nothing to report.

## Conflicts of Interest

The authors declare no conflicts of interest.

## Data Availability

All data generated during this study are available from the corresponding author on reasonable request.
